# CircRNAs in colorectal cancer: potential roles, clinical applications, and natural product-based regulation

**DOI:** 10.3389/fonc.2025.1525779

**Published:** 2025-01-29

**Authors:** Jingjing Yang, Qiqi Fan, Yiyun Wang, Yuanyue Liu, Xiaoning Xu, Yeqi Liang, Jiakang Xie, Jiajie Li, Fengting Ai, Yong Cao, Shangzhen Yu, Jinman Liu

**Affiliations:** ^1^ Central Laboratory, Affiliated Jiangmen Traditional Chinese Medicine (TCM) Hospital of Ji’nan University, Jiangmen, China; ^2^ Department of Oncology, Huangpu People’s Hospital of Zhongshan, Zhongshan, China; ^3^ Science and Technology Innovation Center, Guangzhou University of Chinese Medicine, Guangzhou, China; ^4^ Department of Neurology, The Second Affiliated Hospital of Nanjing University of Chinese Medicine, Nanjing, China; ^5^ School of Traditional Chinese Medicine, Ji’nan University, Guangzhou, China

**Keywords:** colorectal cancer (CRC), circular RNAs (circRNAs), biological function, prognostic biomarkers, potential therapeutic biomarkers, natural product-based regulation

## Abstract

Colorectal cancer (CRC) is one of the leading causes of cancer-related mortality worldwide. In recent years, circular RNAs (circRNAs), a novel class of non-coding RNA molecules, have emerged as a research focus due to their unique stability and functional roles. CircRNAs regulate tumor-related signaling pathways through interactions with microRNAs (miRNAs) and proteins, playing key roles in tumorigenesis, progression, invasion, metastasis, and chemoresistance. This review summarizes the role of circRNAs in CRC, particularly their mechanisms in cell proliferation, migration, apoptosis, tumor microenvironment (TME) remodeling, and immune evasion. Aberrant expression of circRNAs holds great potential as diagnostic and prognostic biomarkers as well as therapeutic targets for CRC. Additionally, natural products such as flavonoids and glycosides, by modulating circRNA-miRNA-mRNA networks, offer promising therapeutic strategies. The article also discusses the current technical challenges in circRNA research and its future application prospects in CRC, highlighting the need for further investigation into the role of circRNAs in tumor immune microenvironments and drug resistance mechanisms.

## 1 Introduction

With 1.14 million emergent cases and an alarming 0.57 million fatalities globally, CRC firmly situates itself as the fifth predominant malignancy in both incidence and mortality (1, 2). It not only pervades as one of the most ubiquitous cancers but also significantly punctuates cancer-related mortality, particularly in Western countries (3, 4). Traditionally, research in CRC has been predominantly tethered to chromosomal instability (CIN) pathways, with APC gene mutations often spotlighted as initial pathogenic events, thereby orchestrating allele loss and somatic gene amplification translocations (5). This conventional carcinogenic model is implicated in approximately 70%-80% of all CRC instances. In the latter decades of the 20th century, a secondary carcinogenic pathway, termed the Mismatch Repair (MMR) or Microsatellite Instability (MSI) pathway, was delineated. This pathway, intricately linked with the inactivation of the MMR gene system, sequentially precipitates the inactivation of mutated tumor suppressor genes (6, 7). The paradigm of this alternative carcinogenic model is Lynch syndrome (8), engendering diploid cancers exhibiting an MSI phenotype (9). However, the 5-year overall survival (OS) rate for CRC is low (10). Consequently, a profound understanding of the fundamental mechanisms underpinning carcinogenesis and tumor progression is imperative to unveil novel diagnostic and therapeutic targets, thereby enhancing the 5-year OS rate for CRC patients. This necessitates a meticulous exploration into the carcinogenesis and molecular foundation of CRC, aiming to identify reliable new biomarkers and therapeutic targets.

The carcinogenesis and evolution of CRC are multifaceted processes, intricately intertwined with both intrinsic (such as genetic mutations and transcriptomic alterations) and extrinsic factors (including dietary influences and Human Development Index (HDI)) (11). Circular RNA (circRNA), a structurally covalent molecular entity, executes both physiological and pathogenic functions, acting as a microRNA (miRNA) sponge, protein translator, or protein decoy (12, 13).

The aberrant expression of circRNAs is progressively acknowledged as a pivotal player in the onset and progression of neoplasms. This investigation delineates the biosynthesis, functionalities, and dysregulation of circRNAs in CRC, forecasting their therapeutic potential as both biomarkers and prospective targets. Conclusively, the study underscores the prevailing limitations requiring attention and envisages forthcoming applications of circRNA in clinical practice.

## 2 CircRNAs

### 2.1 Classification

CircRNAs are generated during transcription through the formation of a 3’-5’ phosphodiester linkage between an upstream 3’splice site (splice acceptor) and a downstream 5’splice site (splice donor) (14, 15). Predominantly, circRNAs are primarily categorized into three principal categories, contingent upon their originating sequences: Exon-intro circRNAs (ElciRNAs), intron-derived circRNAs (ciRNAs), and exonic circRNAs (ecRNAs) (16–18). EIciRNAs and ciRNAs typically reside in the nucleus and are implicated in transcriptional regulation through interactions with the Pol II transcription machinery and U1 snRNP, respectively (19, 20). Conversely, EcRNAs predominantly localize in the cytoplasm, where they can modulate gene expression by acting as miRNA sponges or interacting with RNA-binding proteins (21, 22). A fourth category of circRNAs, known as intergenic circRNAs, has recently been identified. These circRNAs primarily originate from the intergenic regions of the genome and consist of two intronic circRNA fragments formed by the reverse variable splicing of the flanking integrated circRNA. Intergenic circRNAs are non-exonic and have gained significant attention due to advancements in RNA sequencing technology and bioinformatics. Unlike linear RNAs, intergenic circRNAs contain two intronic fragments that are flanked by GT-AC splicing signals, which act as loop-linked splice donors and acceptors. Sequence analyses have indicated that intergenic circRNAs generally exhibit weak conservation. Despite this, knowledge about these circular transcripts and the specific mechanisms regulating circRNA biogenesis is still limited. Some research has provided insight into their potential roles. For instance, a study identified an intergenic circRNA, hsa_circ_0007379, which is downregulated and acts as a novel tumor suppressor in CRC (23). The diverse localization and functionalities of these circRNA classes underscore their potential multifaceted roles in cellular processes and disease states, including CRC.

### 2.2 Biogenesis

Originally deemed as inconsequential, low-abundance splicing byproducts (18), circRNAs have been catapulted into scientific prominence with advancements in sequencing technologies and analytical methodologies, revealing that myriad genes are capable of generating circRNAs, with a single gene often producing diverse circRNAs through alternative splicing cycles. The biogenesis of circRNAs often hinges on non-canonical splicing patterns within pre-mRNAs.

**Figure 1:**
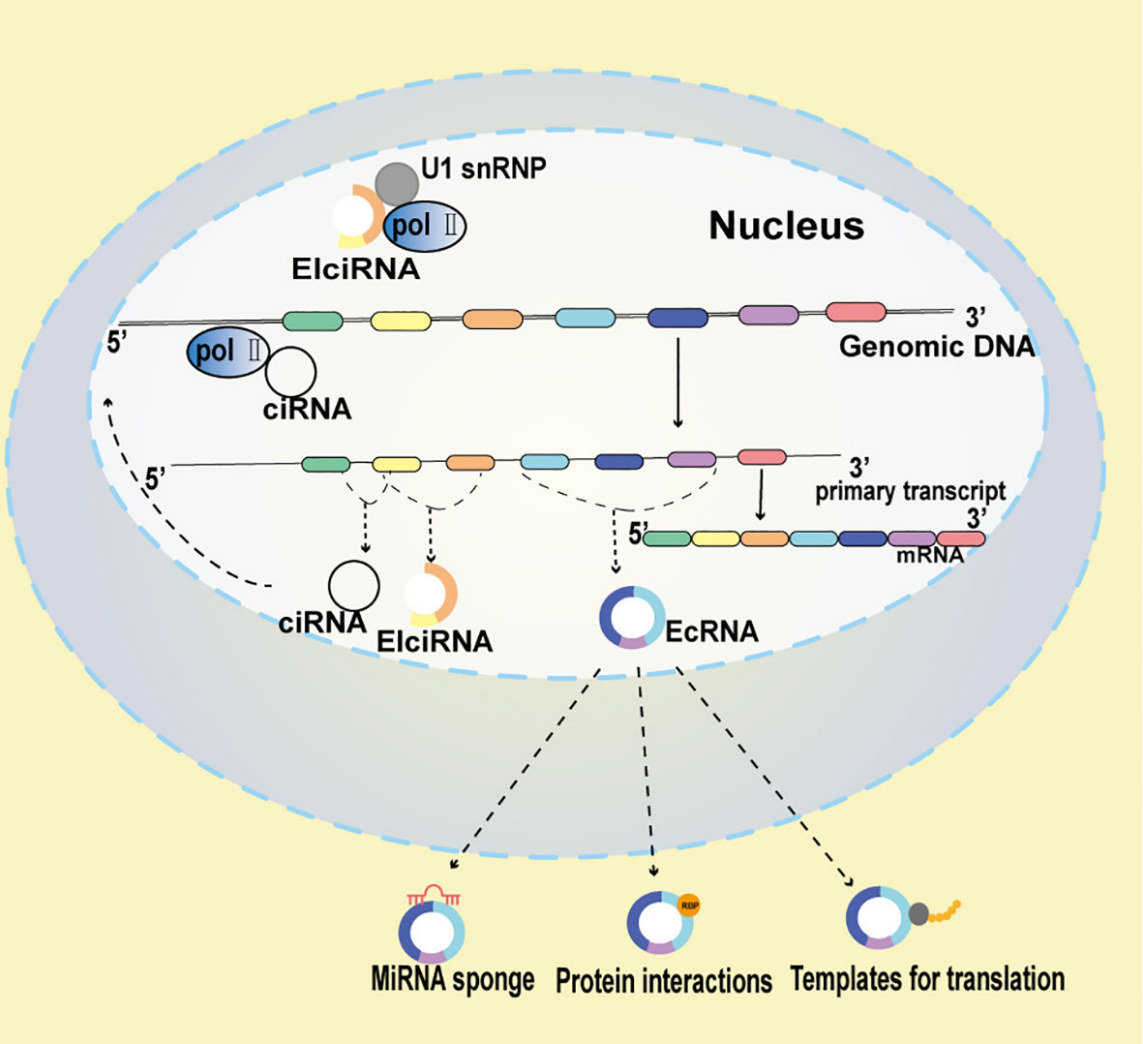
Multifaceted Mechanisms of circRNA function Functionality in CRC. EIciRNAs potentiate the transcription of their parent genes through strategic interactions with Polymerase II (Pol II) and U1 small nuclear ribonucleoproteins (snRNP). ciRNAs, to amplify the transcription of their host genes, directly associate with Pol II. circRNAs can function as miRNA scavengers, mitigating miRNA activity. Additionally, circRNAs may act as molecular decoys, modulating protein behavior, and in certain contexts, they might encode polypeptides.

ElciRNAs interact with U1 snRNP, predominantly localize within the nucleus, and facilitate the transcription of their parental genes. Exon-intron circRNAs, or ElciRNAs are characterized by the retention of introns between circularized exons. The majority of eukaryotic circRNAs, known as ecircRNAs, are formed by exonic sequences. EIciRNAs (**Figure 1**), which are made up of both intronic and exonic sequences, and ecRNAs (**Figure 1**), consisting of exonic sequences, are produced through back splicing in the nucleus. Additionally, since ciRNAs (**Figure 1**) are formed from introns, a typical intron may be modified to become one. The prevailing theory posits that eukaryotic tRNA splicing endonuclease (TSEN) splicing is orchestrated by a ruler mechanism, whereby splice sites are selected according to their proximity to the recognized tRNA body (24). Subsequent to TSEN cleavage, tRNA halves are conjoined to form a mature tRNA. The RtcB enzyme, essential for tricRNA ligation, ligates tRNAs, underscoring the requisite nature of RtcB’s ligase activity. During pre-tRNA maturation, TSEN components cleave pre-intron-containing pro-tRNAs in the conventional bulge-helix-bump (BHB) motif. The resultant intron ends are ligated by RtcB ligase, culminating in the formation of tricRNA, a stable circRNA (25).

### 2.3 Degradation

Compared to mRNA, circRNA exhibits a notably resilient structure. Presently, three principal processes are posited to orchestrate circRNA degradation (26). Firstly, the circRNA-miRNA axis, which dismantles certain circRNAs by leveraging highly complementary binding sites for specific microRNAs (miRNAs), for instance, miR-671 directs the degradation of CDR1as. Secondly, circRNA degradation mediated by N6-methyladenosine (m6A) modification emerges as another pivotal mechanism (27). YTHDF2 (27) interacts with RNase P/MRP (mitochondrial RNA processing) to recognize circRNAs containing the m6A gene through heat-reactive protein 12 (HRSP12) bridging. Additionally, exosomes or extracellular vesicles may be used to remove circRNAs. For instance, exosomes encompass molecules like circSCMH1 and MeCP2 that exhibit persistent presence. Thirdly, MDM2, instrumental in quelling apoptosis by inhibiting p53, Foxo3, and their downstream molecule Puma, plays a crucial role. During cancer cell apoptosis, the circ-Foxo3 expression has been observed to markedly escalate. Consequently, the down-regulation of circ-Foxo3 impedes cancer cell apoptosis (28, 29). For example, the formation of the circ-Foxo3-p21-CDK2 ternary complex arrested the function of CDK2 and blocked cell cycle progression (30). Deng et al. noted a diminution of circ-FOXO3 in tumor tissues, and the overexpression of LATS543 precipitated apoptosis in CRC cells (29).

### 2.4 Stability

The stability of circRNA is mainly reflected in its unique circular structure and its ability to resist degradation. Circular RNA forms a closed loop structure through the reverse splicing mechanism, which makes it unaffected by RNA exonuclease, thus having high stability and not susceptible to degradation. (31)Specifically, due to the lack of a 5 ′ cap and 3 ′ poly (A) tail, circRNA is generally resistant to exbonuclease attack and shows higher stability than homologous linear mRNA with the same nucleotide sequence (32). This property provides the circular RNA with a longer cellular half-life and higher stability, which is crucial for the durable effect of the drug. Moreover, the stability of circular RNA is also manifested in its resistance to nucleases (33). Due to its circular structure, the circular RNA is resistant to degradation by nucleases, maintaining its integrity. This stability allows circular RNA to exist in living organisms for long periods, participate in various biological processes, and has potential applications in disease diagnosis and treatment. (21) Because the internal translation of circRNA is generally inefficient compared to classical cap-dependent translation of linear mRNA, detection of protein and peptide products produced by circRNA is challenging due to technical limitations.

### 2.5 Function

CircRNAs are now recognized to affect the expression of parental genes, function as miRNA sponges, interact with proteins, serve as miRNA sponges, and contribute to the onset and progression of diseases (34).

#### 2.5.1 MiRNA sponge

One of the most important roles of circRNAs is serving as miRNA sponges. CircRNAs harbor binding sites for miRNAs and, by associating with them, can modulate the expression of downstream genes, thereby exerting pertinent biological effects (35). Numerous studies have found that certain circRNAs, such as CDR1as and circPVT1 exhibit miRNA sponge activities (36). Moreover, recent investigations have demonstrated that individual circRNAs can act as sponges for different miRNAs and, depending on the context, either suppress tumors or act as oncogenes. For example, circHIPK3 has been shown to interact with 9 miRNAs through 18 putative binding sites, as determined by a luciferase screening test (37).

#### 2.5.2 CircRNAs-protein interactions

Through interactions with various proteins, circRNAs are able to perform their second crucial role. For example, excessive production of circ-Foxo3 suppressed MDM2’s ability to control Foxo3’s poly-ubiquitination and reduced the interaction between Foxo3 and MDM2 (38). Consequently, Foxo3 activity increased, encouraging Puma expression and cell death. Additionally, circPTK2’s interaction with PABPC1 might particularly promote SETDB1 expression and the SETDB1-mediated epithelial-mesenchymal transition (EMT), which would improve PABPC1’s capacity to stabilize PABPC1 mRNA. Nonetheless, further research is urgently needed to decipher the underlying mechanisms and validate circRNA-mediated protein interactions (39).

#### 2.5.3 Templates for translation

In recent years, circRNAs have been recognized as a group of noncoding RNAs acting as post-transcriptional regulators (40, 41). A recent study has confirmed the translation of multiple circRNAs, involving internal ribosome entry sites (IRESs) (42). These RNAs, capable of being translated into peptides or proteins, have been substantiated by various experiments. An open reading frame (ORF), a subset of circRNA with an initial codon site, and internal ribosome entry site (IRES) elements can be translated in specific situations, resulting in unique peptides with specific functions. The first translated circRNA found in CRC is hsa_circ_0000423, also known as circPPP1R12A, which is expressed at significantly higher levels in colon cancer tissue (43). Within circPPP1R12A, researchers identified an ORF encoding circPPP1R12A-73aa, a functional protein. Further investigations affirmed that circPPP1R12A-73aa, but not circPPP1R12A, increased CRC proliferation, migration, and invasion by stimulating the Hippo-YAP signaling pathway (16). Uncovering their mechanisms could pave the way for a new epoch in CRC diagnostics and treatment. However, the effect of the protein produced by circRNAs on the development and progression of CRC remains to be elucidated.

## 3 Dysregulated expression of circRNAs in CRC

Despite emerging evidence (44, 45), the mechanisms underlying the dysregulated expression of circRNAs in carcinogenesis and CRC development remain unclear. However, several novel findings, broadly characterized as aberrant gene activities such as chromosomal translocations and somatic copy number variations, may provide potential theories. Previous studies have described numerous circRNAs, including aberrant circSTK3 (46) expression, which initiates epithelial-mesenchymal transition programming and alters a group of genes associated with CRC metastases. Moreover, the transcription of circSTK3 is controlled by CTCF (CCCTC binding factor), shedding light on the functional and predictive aspects of circSTK3 and the involvement of circRNAs in metastasis. CircRNAs wield a pivotal role in cancer pathogenesis. For example, we discovered that has_circ_0001681 (47) was significantly down-regulated in glomerular basement membrane (GBM) yet up-regulated in four cancer types (CRC, LUAD, GC, and THCA). Several circRNAs are dysregulated in CRC cells compared to adjacent normal mucosa tissues, as per bioinformatic analyses, suggesting that these dysregulated circRNAs likely harbor significant functional roles in CRC progression.

## 4 Role of circRNAs in CRC

CRC frequently manifests in conjunction with aberrant circRNAs, which can exhibit either oncogenic or suppressive characteristics, thereby altering cellular processes. These circRNAs can regulate immune evasion, reshape the tumor microenvironment (TME), develop multidrug resistance, and affect cell proliferation, migration, and death.

### 4.1 CircRNAs involved in cell proliferation

The disruption of the canonical cell cycle and the presence of enhanced proliferative capabilities are considered the mainstays of tumorigenic pathways (48). Tumorigenesis is predominantly characterized by the activation of oncogene and the inactivation of tumor suppressor genes, which result in aberrant cell cycle. Recently, several circRNAs have been found to support the biological action of cyclins or CDKs in CRC. For instance, Circ_001621 attenuated the restraint of matrix metallopeptidase 9 (MMP9) and cycling-dependent kinase 4 (CDK4) by miR-578, thereby increasing osteosarcoma proliferation and migration, respectively (49). By regulating the balance of glycine/serine metabolism and redox in a p53-dependent manner, circMYH9 overexpression encourages CRC growth (50). CircPPP1R12A influences the Hippo-YAP pathway in CRC cells, enhancing the cells’ ability to proliferate, migrate, and invade (43). Conversely, overexpression of circPIP5K1A promotes CRC cell invasion and migration, while its diminution curtails cell viability and motility (51).

Certain circRNAs modulate CDK or cyclin activity and regulate cell cycle-specific genes to promote cellular growth (52–54). Abnormally produced circRNAs in malignancies may influence cancer cell proliferation by controlling key cell cycle regulators such as cyclins, p53, CDKs, CDK inhibitors (CKIs), and pRB. CircRNAs also affect cell proliferation by forming complexes with factors involved in the cell cycle, controlling CCN through RNA-binding proteins, and regulating major Wnt/β-catenin, phosphatidylinositol-3 kinase (PI3K)/AKT/FOXO, and other signaling pathways (55).

Moreover, circRNAs can affect CRC’s invasion, tumor lymph node metastasis stage, malignant phenotypes, histological grade, and chemotherapy treatment susceptibility by influencing the aforementioned cell cycle variables.

### 4.2 CircRNAs in tissue invasion and metastasis

**Figure 2:**
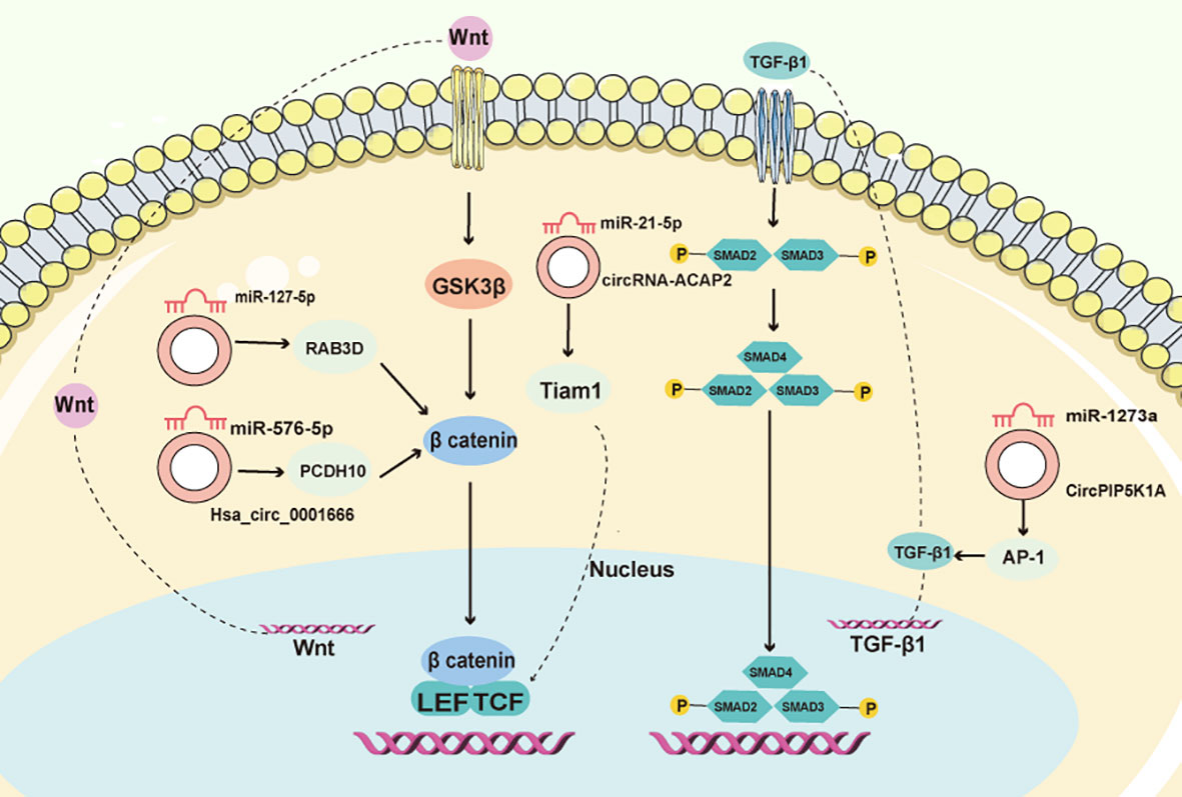
CircRNAs control the CRC EMT process. CircRNAs, through interactions with miRNA, functionally integrate into the TGF-/Smad and Wnt/β-catenin pathways, orchestrating the EMT process in CRC.

Exosomes, diminutive small extracellular vesicles, play a pivotal role in tumor spreading by carrying specific information between cells. They have been found to contribute to various processes involved in cancer progression, such as the epithelial-to-mesenchymal transition (EMT), extracellular matrix (ECM) remodeling, tumor angiogenesis, immune evasion, and the establishment of pre-metastatic niches. Exosomes can also influence organotropic metastasis, which refers to the preference of cancer cells to metastasize to specific organs (39). For instance, in CRC, the circRNAs Hsa_circ_0001666 has been found to decelerate disease progression by modulating the miR-576-5p/PCDH10 axis (56). Moreover, it diminishes EMT and stemness in CRC cells, while also affecting the Wnt/β-catenin signaling pathway (**Figure 2**). Another circRNA, circRNA-ACAP2, is expressed in elevated levels in CRC tissues compared to normal tissues. The knockdown of circRNA-ACAP2 inhibits the proliferation, migration, and invasion of SW480 cells by upregulating miR-21-5p and inhibiting Tiam1, a downstream target of miR-21-5p (57) (**Figure 2**). Additionally, circPIP5K1A has been found to promote colon oncogenesis by inhibiting miR-1273a (51) and amplifying AP-1 activity (**Figure 2**). Furthermore, hsa_circ_0006732 may act as an oncogene in CRC by altering the miR-127-5p/RAB3D axis, influencing CRC cell proliferation, migration, invasion, and EMT (58) (**Figure 2**).

### 4.3 CircRNAs involved in cell apoptosis

CircFoxo3 is a well-studied circRNA derived from the tumor suppressor gene Foxo3 (28), has been implicated in various roles across the development of numerous cancer types. For instance, by boosting FOXO3a expression, ANGPTL1 significantly reduces the expression of the stem cell transcription factor SOX2 in CRC cells, thereby attenuating the cells’ migratory, invasive capabilities, expression of cancer stem cell (CSC) markers, and sphere-forming ability (59, 60). Additionally, ALKBH5, modulating the FOXO3/miR21/SPRY2 axis in CRC, acting as an anticancer agent (61–63). In CRC tissue, circZNF609 expression is downregulated, and it fosters apoptosis by upregulating p53 in CRC cells (64). Furthermore, the disruption of circAPLP2 slows glycolysis *in vitro* and mitigates tumor growth *in vivo* by elevating miR-485-5p and diminishing FOXK1 (65).

### 4.4 CircRNAs involved in angiogenesis

**Figure 3:**
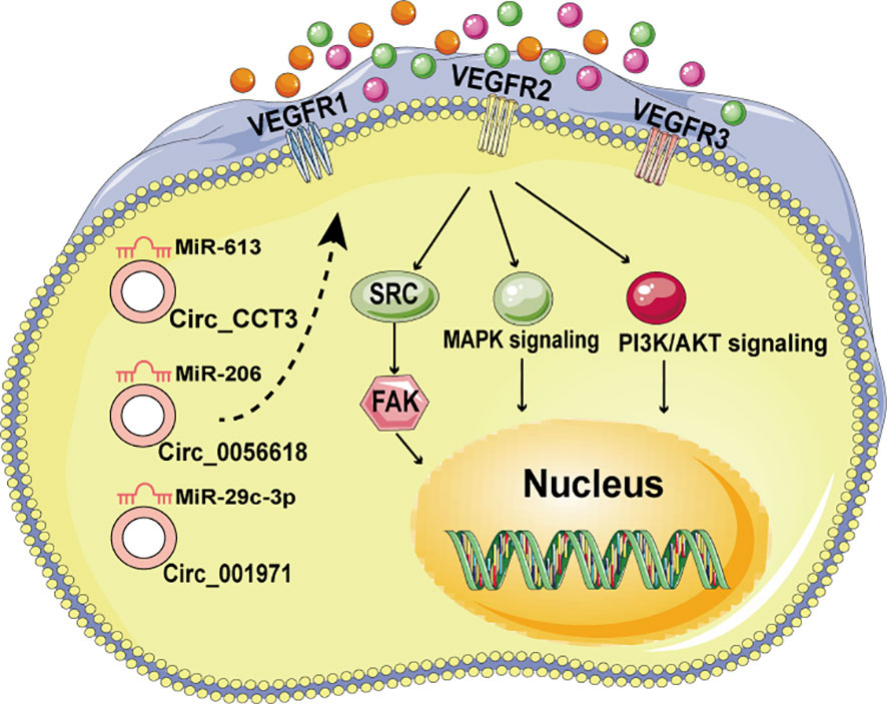
The circRNA/VEGF signaling axis in CRC.

Neovascularization is pivotal in the growth of malignancies, enabling distant metastasis and facilitating nutrient supply and waste removal as tumors attain substantial size (66). Certain circRNAs have been found to promote angiogenesis by modulating angiogenic regulators such as vascular endothelial growth factor (VEGF). For instance, VEGFs interact with tyrosine kinase cell receptors (VEGFR1-3) to activate VEGF signaling in endothelial cells, thereby influencing cell proliferation, migration, and vascular permeability during angiogenesis (67). In CRC, CircCCT3 has been shown to promote CRC metastases through the miR-613/WNT3 or miR-613/VEGFA axis (68, 69) (**Figure 3**). Additionally, Circ_001971 acts as a competing endogenous RNA (ceRNA) to counteract miR-29C-3p’s suppression of VEGFA, thereby enhancing CRC growth, invasion, and angiogenesis (70) (**Figure 3**). Moreover, Circ_0056618 upregulates CXCR4 and VEGF-A in CRC, promoting cell proliferation, migration, and angiogenesis by sponging miR-206 and eliminating its repressive effects (70, 71) (**Figure 3**).

### 4.5 CircRNAs involved in cellular energetics

Glycolysis, pivotal for ensuring an efficient energy supply, particularly in low-oxygen environments, involves key glycolytic enzyme called HK2, which catalyzes the conversion of glucose to glucose-6-phosphate (72, 73). Overexpression of HK2 promoted cell invasion and migration by increasing glycolysis. In CRC, the downregulation of Circ-PITHD1 inhibited CRC invasion and proliferation by regulating miR-590-5p/HK2 signaling-mediated aerobic glycolysis. In certain cancers, hypoxia-induced circRNF13, mediated by HIF-1 and EIF4A3, promotes tumor development and glycolysis (74–76). Hsa_circ_0000231, which suppresses cell glycolysis and promotes cell death, exhibited diminished when hsa-502-5p inhibitor was utilized (77, 78). However, further study is urgently required to elucidate the role of circRNA in cellular energetics of CRC.

### 4.6 Immune destruction of CircRNAs

The PD-1/PD-L1 pathway, an immunological checkpoint, assists tumor cells in evading the immune system, thereby emerging as a compelling target for antitumor immunity (79). Delivery of circRERE-AAV causes significant anticancer effects in preclinical models of CRC, and a combination therapy utilizing circRERE-AAV and an anti-PD-1 antibody exhibits synergistic effects on tumor formation (80). By increasing the expression of PD-L1, hsa_circ_ 0136666 (81), hsa_circ_ 0020397 (82), CDR1-AS (83), and circEIF3K (84) promotes the carcinogenesis and immune evasion of CRC, potentially providing new insights into the molecular immunopathogenesis of CRC. Further studies are needed to better understand the role of noncoding RNAs in tumor immunotherapy and the cancer immune response.

### 4.7 Drug resistance in CRC

The prevalence of drug resistance is notably high, significantly diminishing the chemotherapeutic efficacy once resistance is established. In CRC tissues and cells, an elevation in miR-31-5p and a downregulation of CircDDX17 and KANK1 have been observed. The upregulation of CircDDX17 increased 5-Fu sensitivity and slowed CRC progression (85). CircDDX17, by sponging miR-31-5p, impedes CRC development and 5-Fu resistance. Conversely, the knockdown of FOXO3 via FOXO3 shRNA restored the doxorubicin sensitivity of doxorubicin-resistant HCT116 DR cells. Conversely, FOXO3 overexpression decreased the doxorubicin sensitivity of HCT116 cells (86). In CRC cells, CircLHFPL2 successfully overcame PIK3CA-mediated MEK inhibitor resistance (87). Additionally, CircRNA_101277 has been found to augment cisplatin resistance by suppressing miR-370, elevating the miR-370 target gene IL-6, and activating the miR-370/IL-6 axis (88, 89).

### 4.8 Tumor microenvironment

The TME encompasses various components, including Cancer-associated endothelial/fibroblast cells (CAEs/CAFs), tumor-related macrophages (TAMs), NK cells, and TEXs (90–93). CAFs, subjected to either normoxic or hypoxic treatment, have been observed to release exosomes under hypoxic conditions (94). CRC cells treated with exosomes from sh-circEIF3K CAFs formed fewer cell colonies compared to those treated with exosomes from sh-NC CAFs. Moreover, patients with low circEIF3K levels exhibited longer median survival times compared to their high-level counterparts. TAMs are key players in tumor immunity and escape within the TME. The results of the ISH experiment revealed that circMERTK was substantially more abundant in CRC tissues than in comparable normal tissues, with its expressions largely overlapping with the macrophage marker CD68. The M2 polarization of macrophages is facilitated by exosomal circRNAs (95, 96). Exosomal circEIF3K may influence PD-L1 expression in CRC (84), while exosomal circUHRF1 promotes TIM3 expression in NK cells, diminishing NK cell functionality. Blocking MALAT-1 in the CCL5/MALAT-1/Snail axis prevented Snail overexpression from causing CCL5-induced migration and invasion, demonstrating the vital of this axis in the DC-dependent cancer development. Cancer cells can mediate immune evasion by using exosomal PD-L1. The function of T cells may be inhibited if TEXs carrying PD-L1 reach the lymphatic system from the blood, preventing immune cells from identifying and eliminating tumor cells. Patients with CRC exhibited elevated expression of hsa-circ0004771 in serum exosomes, suggesting its potential future utility as a CRC biomarker (97).

## 5 CircRNAs as potential biomarkers in CRC

Owing to their physically closed circular structure, circRNAs exhibit enhanced stability compared to their linear RNA counterparts (98, 99). Moreover, aberrant expression of circRNAs in CRC has been associated with carcinogenesis and the development of cancer cells (100). Dysregulated circRNAs, bearing a significant correlation with CRC, are posited to hold clinical importance as biomarkers. Here is a description of the potential of circRNAs as diagnostic biomarkers, prognostic biomarkers, and therapeutic biomarkers in CRC.

### 5.1 Diagnostic biomarkers of circRNAs in CRC

The early detection of CRC is pivotal for effective treatment and is often associated with successful outcomes (84, 101). Given their distinct expression patterns in CRC tissues and blood, compared to healthy controls, circRNAs emerge as promising candidates for tissue or liquid biopsies aimed at diagnosing CRC. For instance, hsa_circ_001978, hsa_circ_105039, and hsa_circ_103627 have been identified as potential novel biomarkers for CRC prediction (102). CircRNA_105039, crucial for augmenting CCND2 expression and promoting the formation of cardiomyocytes formation through miR17 absorption, along with circ-FMN2, circ-LMNB1 (103), and circ-ZNF609 may serve as viable diagnostic and prognostic markers for CRC identification. Hsa_circ_0026416 may act as a ceRNA by upregulating NFIB expression by competitively absorbing miR-346 (104). Circ_PVT1 and circ_001569, exhibiting significant upregulation in individuals with CRC, suggest their potential utility as diagnostic and prognostic marker for CRC (105). Hsa_circ_0007534 may serve as a potential cancer marker in CRC patients and might be associated with adverse prognostic outcomes (106). Furthermore, the diminished expression of hsa_circ_0001649 in CRC suggests its potential utility as a biomarker for targeted and sensitive CRC screening (107).

### 5.2 Prognostic value of circRNAs in CRC

Intervening with prognostic variables is imperative to enhance clinical outcomes and potentially facilitate curative approaches in CRC (108–110). CircRNA_0001178 and circRNA_0000826 have emerged as potential diagnostic biomarkers for liver metastases originating from CRC (111, 112). MiR-7, considered a tumor suppressor in CRC, inhibits the expression of numerous oncogenes and has been implicated in tumor suppression through combined circHIPK3 silencing and miR-7 overexpression, as evidenced by *in vitro* and *in vivo* studies (113, 114). CircLONP2, acting as a pivotal initiator of metastasis during CRC progression, influences miR-17 intracellular maturation and intercellular transfer (115). Circ_382390 inhibits miR-30c-5p, which increases MYC and CCND1 in the target gene TCF7 (116). CircHUWE1, potentially sponging miR-486, may counteract the tumor-suppressive effects of silence when miR-486 is downregulated (117). Additionally, both hsa_circ_0052184 (118) and CircVAPA have been implicated in promoting CRC proliferation and migration (119). Circ_0021977, acting through the circ_0021977/miR-10b-5p/p21&p53 axis, presents itself as a potential target for CRC therapy, influencing invasion (120).

### 5.3 Potential therapeutic role of circRNAs in CRC

CircRNAs have emerged as potential therapeutic targets in CRC, with over 70 elevated circRNAs exerting a tangible impact on CRC carcinogenesis. The silencing of these circRNAs yields antithetical effects both *in vitro* and *in vivo*, positioning these oncogenic circRNAs as viable therapeutic targets. Specially created targeted interference RNAs can precisely target special post-splicing ligations of cancer-causing circRNAs for anti-tumor effects. For instance, circ-RNF121 absorbs miR-1224-5p and associates with FOXM1, thereby inhibiting tumor development *in vivo* (121). Additionally, circ-133 (122) and hsa_circ_0005963 (74) have been identified as therapeutic biomarkers in CRC. CircIFT80, by sponging miR-634, miR-568, and miR-142, and its circIFT80’s positive connection with CTNNB1 (β-catenin), augments gene expression (123). The circFNDC3B-miR-97-5p-TIMP3 pathway has demonstrated tumor-suppressive activity, and circFNDC3B-enriched exosomes have been shown to inhibit angiogenesis and CRC progression (124). CircCOG2, when upregulated, accelerates CRC proliferation and invasion via the miR-1305/TGF-2/SMAD3 pathway (125). Exosomes derived from CRC cells with high metastatic potential could spread this effect to CRC cells with lower metastatic potential. Circ_0006174, an oncogenic circRNA, modulates the miR-1205/CCBE1/Wnt pathway, facilitating the progression of CRC (126) (**Table 1**). It is ensured that all referenced studies have been approved by the respective ethics committees and adhere to all necessary ethical standards, all participants signed an informed consent form.

**Table 1:** Main biomarkers in CRC.

Biomarker type	CircRNAs	Expression	Biological function or role	Reference
Diagnostic biomarker	Hsa_circ_001978	↑	Promoting the proliferation and invasion of CRC; sponging for miR-134; AUC = 0.966.	(102)
	Hsa_circ_105039	↑	Promoting the proliferation and invasion of CRC; sponging for miR-134; sponging for miR‐17; AUC = 0.966.	(102)
	Circ-FMN2	↑	Promoting the proliferation and invasion of CRC; AUC =0.9153.	(103)
	Circ-LMNB1	↑	Promoting the proliferation and invasion of CRC; AUC =0.9627.	(103)
	Circ-ZNF609	↑	Promoting the proliferation and invasion of CRC; AUC =0.8711.	(103)
	Hsa_circ_0026416	↑	Promoting the proliferation and invasion of CRC; sponging for miR-346; AUC =0.767.	(104)
	Circ_001569	↑	Promoting the proliferation and invasion of CRC; AUC =0.9016.	(105)
	Circ_PVT1	↑	Promoting the proliferation and invasion of CRC; AUC =0.8389.	(105)
	Hsa_circ_0007534	↑	Promoting the proliferation and invasion of CRC; AUC =0.780.	(106)
	Circ_0001649	↓	Promoting the proliferation and invasion of CRC; AUC =ROC 0.857.	(107)
Prognostic biomarker	CircVAPA	↑	Promoting the proliferation, invasion, migration of CRC.	(119)
	CircHUWE1	↑	Promoting the proliferation, invasion, migration of CRC; sponging for miR-486.	(117)
	Hsa_circ_0052184	↑	Promoting the proliferation, invasion, migration of CRC.	(118)
	Circ3823	↑	Promoting the proliferation, invasion, migration of CRC; sponging for miR-30c-5p.	(116)
	CircLONP2	↑	Promoting the proliferation, invasion, migration of CRC; sponging for miR-17.	(115)
	Circ_0021977	↓	Promoting the proliferation, invasion, migration of CRC; sponging for miR-10b-5p.	(120)
Therapeutic biomarker	Circ_133	↑	Therapeutic target by binding to circ_133a.	(122)
	Circ_0005963	↑	Therapeutic target by binding to miR-122.	(74)
	CircIFT80	↑	Therapeutic target by sponging miR-142, miR-568, and miR-634 upregulated the gene expression.	(123)
	CircFNDC3B	↓	Therapeutic target through circFNDC3B-miR-97-5p-TIMP3 pathway.	(124)
	CircCOG2	↑	Therapeutic target through the miR-1305/TGF-β2/SMAD3 pathway.	(125)
	CircRNF121	↑	Therapeutic target by promoting FOXM1expression to regulate the proliferation and invasion sponging miR-1224-5p and miR-1224-5p.	(121)
	Circ_0006174	↑	Therapeutic target through the miR-1205/CCBE1/Wnt pathway.	(126)

↑ means the expression of the gene is upregulated.

↓ means the expression of the gene is downregulated.

### 5.4 Applications in targeted therapy

The decision to use targeted therapies has traditionally been based on histopathological features and genotyping. However, only a small proportion of patients benefit from these approaches, highlighting the need for more refined biomarkers, particularly those that indicate specific sensitivity to targeted drugs. Next-generation sequencing and high-throughput technologies have significantly advanced biomarker discovery and enhanced our understanding of the underlying mechanisms of targeted therapies.

Due to its short half-life and significant fluctuations in expression under different physiological conditions, mRNA is not an ideal predictor for targeted therapy responses. In contrast, circRNAs offer advantages over linear mRNA due to their stable, circular structure, which confers significant resistance to degradation and greater stability (127).

Anlotinib (128) is a common targeted agent used in cancer treatment. It inhibits tumor growth *in vitro* and *in vivo* by downregulating circHAS2. These findings suggest a promising strategy for targeting patients with advanced colorectal cancer (CRC) who exhibit higher levels of circHAS2, potentially benefiting approximately 52.9% of such patients. In parallel, a prospective phase II clinical study has been initiated to evaluate the drug’s efficacy in 14 patients with advanced CRC (ClinicalTrials.gov identifier: NCT05262335). Although recent guidelines do not yet include anlotinib, circRNAs may offer valuable guidance in future studies of targeted drugs.

Cetuximab (129) is another targeted therapy with excellent efficacy in treating CRC, particularly in metastatic cases. It is widely used as a first-line treatment for CRC. Despite its promising clinical benefits, including improved progression-free survival (PFS), overall survival (OS), and quality of life with minimal side effects, the effectiveness of cetuximab is often limited by chemoresistance. Dysregulation of circRNA expression in CRC, breast cancer, and non-small cell lung cancer (NSCLC) has been linked to resistance to chemotherapy and targeted therapies. However, this resistance can often be reversed by modulating circRNA expression in tumor cells.

For example, cetuximab resistance in CRC has been associated with mutations in RAS, BRAF, PIK3CA, and PTEN. A study by Zhang et al. (130) demonstrated that circIFNGR2 regulates the target gene KRAS through a miR-30b sponge mechanism at the post-transcriptional level, thereby contributing to cetuximab resistance in CRC cells. In contrast, inhibiting circHIPK3 (113) expression enhances the responsiveness of CRC cells to cetuximab. Moreover, a retrospective analysis revealed that circHIF1A-positive patients had lower tumor regression rates and poorer long-term prognosis compared to circHIF1A-negative patients. The expression of circHIF1A in tumor tissues may serve as a predictive marker for the efficacy and outcomes of cetuximab treatment in RAS/BRAF wild-type metastatic CRC (mCRC) patients (129).

Programmed cell death ligand 1 (PD-L1) is a critical immune checkpoint protein that regulates immune responses through its interaction with PD-1. Knockdown of circ_0007422 (131) has been shown to suppress tumor progression and immune escape in CRC by modulating the miR-1256/PD-L1 pathway, offering a novel therapeutic approach for CRC. Additionally, circ_0089761 (132) competes with PD-L1 for binding to miR-27b-3p, relieving miR-27b-3p’s inhibitory effect on PD-L1 expression. This results in increased PD-L1 levels, promoting metastatic capacity in CRC cells. Therefore, the circ_0089761/miR-27b-3p/PD-L1 axis may serve as an important therapeutic target for CRC treatment. Elevated levels of circ_0089761 in CRC cells contribute to the upregulation of PD-L1, further enhancing tumor progression.

### 5.5 CircRNA abundance in clinical prognosis

An increasing body of research has highlighted the dysregulation of circRNA expression in the progression of CRC, underscoring its critical role in the development of these cancers. Previous studies have shown that circRNAs not only act as miRNA sponges in the progression of intestinal cancers but also modulate drug sensitivity. Furthermore, many circRNAs have been identified as potential prognostic markers for the early diagnosis and prognosis of CRC, owing to the stability provided by their unique circular structure.

Current blood-based biomarkers, such as CEA and CA19-9, suffer from low sensitivity and specificity. Additionally, other diagnostic methods are either invasive or costly. As a result, recent research has shifted toward minimally invasive testing, particularly liquid biopsy. Exosomes, which are abundant, stable in circulation, and rich in genetic information and other biomolecules, are emerging as key molecules for biomarker discovery. These exosomal non-coding RNAs, including miRNAs, lncRNAs, and circRNAs, have shown promise in early CRC detection, offering higher sensitivity and specificity than traditional biomarkers like CEA and CA19-9. Moreover, exosomal circRNAs have demonstrated significant prognostic potential for TNM staging and could serve as predictive biomarkers for chemotherapy regimens such as 5-FU and FOLFOX.

CircLPHN3 has been shown to be closely correlated with various clinical features of CRC. The expression level of circLPHN3 in cancer tissues negatively correlated with CRC prognosis in Kaplan-Meier survival analysis. MiR-142-5p, which is highly expressed in CRC, is negatively regulated by circLPHN3. Overexpression of circLPHN3 suppresses CRC cell growth, migration, and invasion, mediated through miR-142-5p (133). In a study involving 100 paired CRC and adjacent normal tissue samples, circXRN2 was found to promote CRC cell proliferation and metastasis via the miR-149-5p/ENC1/EMT axis, suggesting that circXRN2 may serve as a potential therapeutic target and a novel biomarker for CRC progression (134).

In a study involving 157 CRC patients and 100 healthy controls, the expression of hsa_circ_0008621 was quantified in both serum and tissue samples by qRT-PCR. The levels of hsa_circ_0008621 were found to be significantly higher in the serum and tissues of CRC patients compared to healthy controls. Kaplan-Meier analysis and multivariate Cox regression indicated that high serum levels of hsa_circ_0008621 are associated with poor survival in CRC patients, promoting tumor progression. These findings suggest that hsa_circ_0008621 could serve as a promising non-invasive prognostic biomarker and therapeutic target for CRC (135).

A recent study also demonstrated that circCOL1A1 was significantly upregulated in the plasma of CRC patients and positively correlated with TNM stage (p = 0.001). Moreover, dynamic monitoring of circCOL1A1 in CRC patients undergoing surgical resection revealed a significant decrease in circCOL1A1 levels post-surgery, while levels increased in patients with recurrent or metastatic CRC. These findings suggest that circCOL1A1 may serve as a prognostic biomarker for postoperative recurrence in CRC (136).

## 6 Active ingredients in natural products targeting circRNAs in CRC

In recent years, natural products have garnered significant attention for their potential to regulate circRNAs in treating CRC. CircRNAs, a novel class of non-coding RNAs, play crucial roles in cancer progression by sponging miRNAs and regulating downstream gene expression. Numerous studies have demonstrated that different natural active compounds can exert anticancer effects by targeting specific circRNAs (**Table 2**). For example, baicalin, derived from *Scutellaria baicalensis*, upregulates miR-761 expression, which suppresses the downstream signaling of HDGF (hepatoma-derived growth factor), exhibiting remarkable antitumor activity (137). Similarly, kaempferol, extracted from *Rhizoma kaempferiae*, inhibits CRC metastasis by modulating the circ_0000345-mediated JMJD2C/β-catenin signaling pathway (138). Likewise, Icariside II, a flavonoid isolated from *Herba epimedii*, regulates circβ-catenin to suppress tumorigenesis by influencing the β-catenin/Wnt signaling axis (139).

**Table 2:** Research on the regulation of circRNAs by active ingredients in natural products in the treatment of CRC.

Active ingredients	Natural product sources	Structural formula	Related circRNAs	Mechanisms of action	References
Baicalin	*Scutellaria baicalensis*	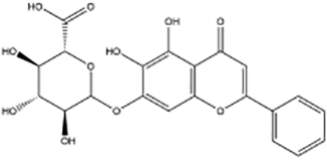	circMYH9	Up-regulation of miR-761 showed antitumor activity in CRC cells by the downregulation of circMYH9 and HDGF	(137)
Kaempferol	*Rhizoma kaempferiae*	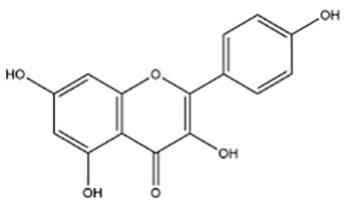	circ_0000345	Inhibition of CRC metastasis via the circ _ 0000345-mediated JMJD2C/β -catenin signaling pathway	(138)
Icariside II	*Herba epimedii*	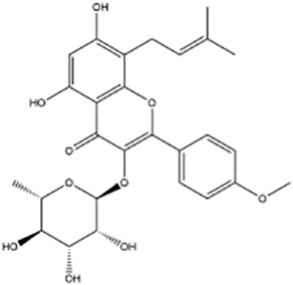	circβ-catenin	Ininhibit tumorigenesis by regulating the circ β -catenin-Wnt/β -catenin axis in colorectal cancer	(139)
Quercetin	*Cacumen biotae*	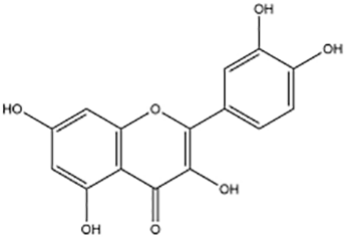	circRNA	Induction of apoptosis in CRC cells via activation of cyclic adenosine phosphate via the circRNA-miRNA-mRNA network	(140)
Aloperine	*Herba sophorae*	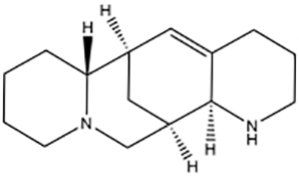	circNSUN2	Inhibition of CRC cell proliferation and promoting apoptosis by modulation of the circNSUN2/miR-296-5p/STAT 3 pathway	(141)
Astragaloside IV	*Astragalus membranaceus*	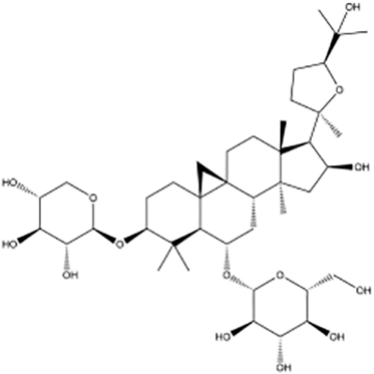	circ_0001615	Development of the CRC, alleviated through the circ _ 0001615/miR-873-5p/LASP 1 pathway	(142)
Ginsenoside Rb1	*Panax ginseng*	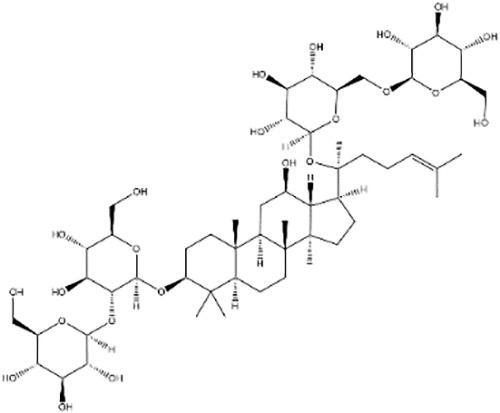	circ-0034880	Reducing circ-0034880 generation by inhibiting the QKI protein shows an anticancer hepatic metastatic potential	(143)
Ganoderic acid Me	*Ganoderma lucidum*	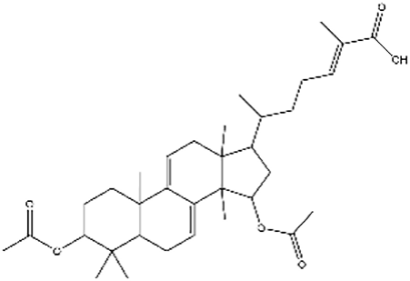	circRNA_07908	Inhibition of CRC cell proliferation and promoting apoptosis by modulation of the circNSUN2/miR-296-5p/STAT 3 pathway	(141)

In addition to flavonoids, other types of natural compounds have shown distinct mechanisms of action. Quercetin, derived from *Cacumen biotae*, activates the circRNA-miRNA-mRNA network to induce apoptosis in CRC cells, demonstrating significant anticancer effects (140). Alopecurine, sourced from *Herba sophorae*, inhibits CRC cell proliferation and promotes apoptosis by targeting circNSUN2 and modulating the miR-296-5p/STAT3 signaling pathway (141). Furthermore, astragaloside IV, extracted from *Astragalus membranaceus*, alleviates CRC progression by acting on the circ_0001615/miR-873-5p/LASP1 axis, highlighting its potential in antitumor therapy (142).

Additionally, ginsenoside Rb1, a major active component of *Panax ginseng*, reduces circ-0034880 generation by inhibiting the QKI protein, showcasing its therapeutic potential against hepatic metastases (143). Lastly, ganoderic acid Me, derived from *Ganoderma lucidum*, inhibits CRC cell proliferation and induces apoptosis through the circNSUN2/miR-296-5p/STAT3 pathway (141). These studies reveal the diverse mechanisms by which different natural compounds regulate circRNA networks and target critical signaling pathways in CRC, thereby exhibiting significant anticancer effects.

In summary, natural products offer unique advantages in circRNA regulation and CRC treatment, opening new avenues for molecular-targeted therapies. These findings provide a scientific foundation for developing precision medicine strategies based on natural compounds. However, further research is needed to explore the clinical feasibility of these mechanisms and evaluate potential off-target effects, aiming to translate these basic research findings into clinical applications effectively.

## 7 Discussion and conclusion

The advent of high-throughput RNA-seq and Ribo-Zero detection techniques has facilitated the identification of a plethora of circRNAs. Intronic circRNAs, such as ciRNAs and EIciRNAs, are postulated to function as transcriptional modulators in the nucleus, given their molecular composition (144, 145). Not only act as sponges for proteins or miRNAs but also have the potential to be translated into proteins or peptides. The predominant focus on circRNAs in tumor progression has been their role as miRNA sponges, potentially overshadowing other mechanisms through which circRNAs might influence tumor progression. Furthermore, previous investigations into the properties and functions of circRNAs have largely omitted exploration into their associations with polymorphic microbiota and senescent cells, both of which are emerging as significant hallmarks of carcinogenesis.

Numerous circRNAs remain unexplored, and their functions, which may be pivotal, warrant further investigation. This gap in knowledge is largely attributed to technological limitations. CircRNAs exhibit biological functions in the development of CRC, predominantly modulating immune destruction, the TME, angiogenesis, cellular energetics, metastasis, apoptosis, and cell proliferation. Furthermore, these circRNAs may serve as universal targets for the detection and treatment of various tumors, laying a robust foundation for pan-cancer research (146). Intriguingly, numerous studies have showed that circRNAs could be dysregulated in various cancers. For example, CiRS-7 have been found to be upregulated in several cancer types (147). Other circRNAs, such as HHLA2 (148), circFADS2 (149), circMYBL2 (150), hsa_circ_0001020 (151), and hsa_circ_0048122 (152), have been proposed as the prognostic biomarkers or potential therapeutic biomarkers of cancer.

While the precise mechanisms through which circRNAs operate in CRC remain elusive, aberrantly expressed circRNAs are progressively emerging as potential biomarkers in diagnosis, prognosis, and therapy. The utility of circRNAs as diagnostic biomarkers is underscored by several key attributes: First, stability: CircRNAs exhibit notable stability and are conserved across species within cells, facilitating their detection (153, 154). Second, specificity: they often demonstrate specific expression across different tissues and developmental stages. Third, presence in body fluids: CircRNAs are discernible in a variety of body fluids, including serum, plasma, and urine.

However, the covalent closure nature of circRNAs presents challenges in their analysis, particularly for specific types. For instance, next-generation sequencing and specialized technologies are imperative for identifying and quantifying platelet-derived circRNAs, rendering accurate detection both costly and time-consuming.

Presently, circRNAs have not been incorporated into clinical settings. The NCI Clinical Trials Registry lists numerous studies on cancer and related complications, but clinical trials involving circRNA are scant. To date, only three circRNA-related cancer clinical trials have been initiated (NCT05934045, NCT05771337, NCT06042842, accessed Jan 23, 2023). All are in phase I/II trials and have yet to report results.

Natural products have shown considerable promise in targeting circRNAs for CRC treatment. Flavonoids, such as baicalin from Scutellaria baicalensis and kaempferol from Rhizoma kaempferiae, regulate circRNAs like miR-761 and circ_0000345, respectively, to suppress tumor growth and metastasis (137, 138). Similarly, Icariside II from Herba epimedii modulates circβ-catenin to influence the β-catenin/Wnt signaling axis, a key pathway in CRC tumorigenesis (139). Other compounds, including quercetin and astragaloside IV, activate circRNA-miRNA-mRNA networks or modulate circRNA-related signaling pathways, inducing apoptosis and inhibiting tumor progression (140, 142). Moreover, ginsenoside Rb1, derived from Panax ginseng, targets circ-0034880, showing promise in preventing CRC hepatic metastasis (143). These natural compounds provide new insights into circRNA-based mechanisms, offering potential therapeutic strategies for CRC by targeting critical tumor pathways. These findings highlight the potential of natural products in targeting circRNAs for CRC therapy. By regulating specific circRNAs, such as miR-761, circ_0000345, and circ-0034880, compounds like baicalin, kaempferol, Icariside II, and ginsenoside Rb1 can influence key tumorigenic pathways, including Wnt/β-catenin signaling. This approach may provide novel therapeutic strategies for CRC, particularly in inhibiting tumor progression and metastasis. However, further clinical studies are essential to confirm the therapeutic potential and safety of these natural compounds in CRC treatment.

## 8 Perspectives

The emerging role of circRNAs in CRC offers promising directions for future research, particularly in elucidating their functions and mechanisms in tumorigenesis and metastasis. As non-coding RNAs, circRNAs possess unique properties such as stability and the ability to regulate gene expression, making them ideal targets for therapeutic intervention. Future studies should focus on understanding the full range of circRNA functions in CRC, including their interactions with miRNAs, mRNAs, and key signaling pathways.

In this context, active ingredients in natural products have also shown potential in regulating circRNAs. For example, baicalin from Scutellaria baicalensis, kaempferol from Rhizoma kaempferiae, and Icariside II from Herba epimedii have been shown to target circRNAs to suppress tumor growth and metastasis in CRC (137, 138). These natural compounds not only modulate circRNA expression but also influence related miRNA and signaling pathways, affecting processes such as apoptosis and the tumor immune microenvironment, thus presenting therapeutic potential for CRC. Future research can further explore how these natural compounds intervene in the multi-step progression of CRC by specifically targeting circRNA-mediated signaling pathways, especially in drug-resistant or metastatic CRC.

Innovative research methodologies, such as high-throughput RNA sequencing and CRISPR technology, will play a crucial role in identifying novel circRNAs involved in CRC progression. Additionally, advancements in single-cell RNA sequencing could help dissect the functions of circRNAs in specific tumor microenvironments, promoting a more precise understanding of their role in CRC heterogeneity.

In terms of translational applications, circRNAs hold great potential as early diagnostic and prognostic biomarkers for CRC. Due to their stability in blood and tissue samples, circRNAs are promising candidates for non-invasive liquid biopsy techniques. Furthermore, targeted therapies aimed at modulating circRNA expression or restoring their normal function could provide new therapeutic strategies, particularly in resistant or metastatic CRC cases.

Overall, circRNA-based therapeutic and diagnostic tools represent an exciting frontier in the broader oncology field, with the potential to revolutionize CRC treatment and personalized medicine. Further exploration of how active ingredients from natural products regulate circRNAs will provide new perspectives for targeted therapies in colorectal cancer.
